# On the usefulness of graph-theoretic properties in the study of perceived numerosity

**DOI:** 10.3758/s13428-021-01733-z

**Published:** 2022-03-29

**Authors:** Martin Guest, Michele Zito, Johan Hulleman, Marco Bertamini

**Affiliations:** 1 University of Liverpool, Liverpool, UK; 2 University of Manchester, Manchester, UK; 3 University of Padova, Padova, Italy

**Keywords:** Numerosity, Graph theory, Principal component analysis, Occupancy model

## Abstract

Observers can quickly estimate the quantity of sets of visual elements. Many aspects of this ability have been studied and the underlying system has been called the Approximate Number Sense (Dehaene, 2011). Specific visual properties, such as size and clustering of the elements, can bias an estimate. For intermediate numerical quantities at low density (above five, but before texturization), human performance is predicted by a model based on the region of influence of elements (occupancy model: Allïk & Tuulmets, 1991). For random 2D configurations we computed ten indices based on graph theory, and we compared them with the occupancy model: independence number, domination, connected components, local clustering coefficient, global clustering coefficient, random walk, eigenvector centrality, maximum clique, total degree of connectivity, and total edge length. We made comparisons across a range of parameters, and we varied the size of the region of influence around each element. The analysis of the pattern of correlations suggests two main groups of graph-based measures. The first group is sensitive to the presence of local clustering of elements, the second seems more sensitive to density and the way information spreads in graphs. Empirical work on perception of numerosity may benefit from comparing, or controlling for, these properties.

## Introduction

Humans and other animals can estimate the quantity of sets of visual elements. This ability has been called the number sense (Cantlon, Platt & Brannon, 2009; Dehaene, 2011). However, estimations can be systematically biased by visual properties, such as size and clustering of the elements. Many studies have investigated the effect of visual properties of 2D configurations of elements on perceived numerosity. In this paper, we focus on various indices from graph theory and their relevance for the perception of numerosity. We will start with a review of the numerosity literature and an introduction to the relevant aspects of graph theory. Then we report a series of analyses. This analysis identifies two main groups of measures; one sensitive to presence of local clustering of elements, the other more sensitive to density.

## Perception of numerosity

The capacity to estimate the difference in quantity or numerosity between two sets of elements without the use of counting or symbolic representation, is an important ability present in humans and in other species (Dehaene, 2011; Neider, 2019). There are two separate mechanisms. For small sets (*N* < 5), the precise number is rapidly determined without the need to individually attend to each item (Kaufman et al., 1949; Trick & Pylyshyn, 1994). For larger sets, when counting or immediate apprehension of quantity is not possible, a different mechanism allows numerosity estimation (Burr & Ross, 2008; Dehaene, 1992; Izard & Dehaene, 2008). This approximate number system (ANS) is responsible for non-symbolic representation of large numerosities, and it is used in basic operations such as estimation, subtraction, and comparison (Cantlon & Brannon, 2007). A feature of the ANS is that with increasing difference in numerosity between two configurations, the task of choosing the larger set becomes easier: the distance effect. Furthermore, if the size of both sets increases, the task of extracting the larger set becomes harder: the size effect. Hence, the most important parameter is the ratio of the two numerosities, in accordance with Weber's law.

Studies of the ANS in humans and non-human animals have found converging results. For instance, mosquitofish can discriminate between social groups if the ratio in group size is at least 1:2 (Agrillo et al., 2008). In non-human primates, numerosity performance decreases when the numerical distance between sets becomes smaller (Barnard et al., 2013). When it is difficult to process separate elements within dense patterns, they are perceived as texture and the properties of the estimation process change (Anobile et al., 2014, 2015).

**Fig. 1:**
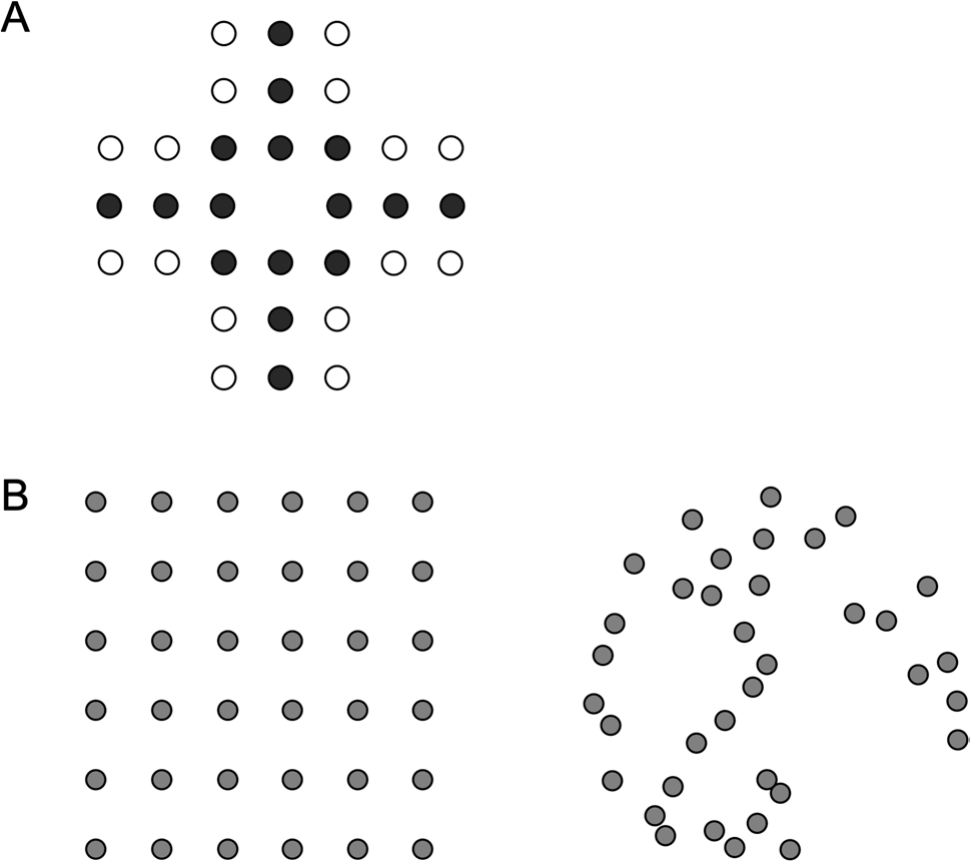
**A** Solitaire illusion. People perceive that there are more black than white dots (Frith & Frith, 1972). **B** Regular-Random Numerosity Illusion. A regular configuration of dots (*on the left*) is perceived as more numerous than the random configuration *on the right*. Both configurations have the same number of dots (Ginsburg, 1976)

It is well known that several properties of the stimuli affect perceived numerosity. In particular, the geometric configuration of the elements biases judgements of numerosity. We can see evidence of this in two phenomena: the solitaire illusion (Frith & Frith, 1972) and the regular-random numerosity illusion (Ginsburg, 1976; Ginsburg, 1980), see Fig. 1. For the solitaire illusion, a regular pattern of black-and-white dots (similar to the pieces in the game of peg solitaire) leads to a striking impression that the dots in the center are more numerous. For the regular-random effect, the elements that are spaced evenly are perceived as more numerous than the randomly distributed elements. In both cases, and also in more generic configurations, it is the groupings or clustering of the items that influences perceived numerosity (Allik & Tuulmets, 1991; Bertamini et al., 2016; Cousins & Ginsburg, 1983; Im et al., 2016).

The observation that grouping leads to underestimation led to the occupancy model (Allik et al., 1991; Allïk & Tuulmets, 1991). According to this model, each element is surrounded by a region of influence whose effect decays with the distance to the element (Burgess & Barlow, 1983). If two or more elements are close together, then their respective regions of influence will overlap, and their combined contribution to the perceived numerosity diminishes. Allïk and Tuulmets (1991) approximated these regions by circles of fixed radius *r*. If two elements are at a distance smaller than 2*r*, then the circles of influence will overlap and their combined area, or occupancy value, will decrease. The model predicts that configurations with a greater occupancy value will be perceived to have greater numerosity.

Both the Solitaire illusion and the Regular-Random Numerosity illusion show the importance of configuration. Frith and Frith (1972) already explicitly mentioned grouping and Gestalt formation. Proximity has a key role in grouping (Kubovy & Wagemans, 1995), and as we have seen the occupancy model provides a measure based on proximity. Proximity may also lead to crowding, as a possible cause of underestimation (Chakravarthi & Bertamini, 2020; Valsecchi et al, 2016). Not everything, however, can be reduced to proximity. If we look at the Solitaire illusion in Fig. 1, for example, the average distance between elements is greater for the white than for the black dots, and yet the black dots are judged as more numerous. This is the opposite of the effect of average distance in the Regular-Random Numerosity illusion. Another curious and unexplained effect occurs when we compute the area of the convex hull. The area is larger for the white compared to the black dots in the Solitaire illusion. Therefore, although in the general case the larger area leads to an increase in perceived numerosity (Hurewitz, et al., 2006), in the case of this particular configuration we have the opposite effect. More work is therefore still necessary to understand the interactions between elements when they are organized in Gestalts or form sub-structures.

## Graph theory

Graph theory is a branch of mathematics that dates back to, at least, Euler (1707–1783) and the famous Königsberg bridges problem (which Euler proved to have no solution). Its main object of study are collections of objects, modeled as dots, points, or vertices, and the relationships between these elements, usually represented as edges (lines connecting pairs of points). In this paper, we use the term vertex for an element, and edge for the line connecting two vertices. We present some graph-theoretic numerical measures that could be useful to capture properties of configurations, and therefore also explain numerosity judgements. We report a correlation analysis between pairs of these measures and between these measures and occupancy because the latter is known as an effective model for perception of numerosity.

A graph *G* can be defined as a finite structure formed by a non-empty set of vertices {*v*_1_, *v*_2_, …, *v*_*n*_} and a set of edges connecting pairs of vertices. Let *G*(*V*, *E*) denote a graph with vertex set *V* and edge set *E*. If {*v*_*i*_, *v*_*j*_} is an edge, denoted as *e*_*ij*_, then both vertices in *e*_*ij*_ are said to be adjacent with each other. The number of edges of a vertex *v*_i_ denotes its degree deg(*v*_i_). Matrices offer an alternative way to describe graphs. Given a graph *G* with *n* vertices the adjacency matrix of *G* is an *n* × *n* table *A*_*G*_ whose rows and columns are indexed by the vertices of *G* and element of ${a}_{v_i{v}_j}$ of *A*_*G*_will have a value of 1 if *v*_*i*_ and *v*_*j*_ are adjacent and 0 otherwise.

A walk, defined as *W* = *v*_0_, *e*_0_, *v*_1_, …, *v*_*f* − 1_, *e*_*f*_, *v*_*f*_ is an alternating list of vertices and edges, where *v*_0_ and *v*_*f*_ are the endpoints of the walk *W* on graph *G*. A walk that has no repeated edges is called a trail, and a walk with no repeated vertex is a path. The only exception to this is if the endpoints are the same vertex, then we have a closed path or cycle. Likewise, a trail whose endpoints are the same vertex is called a closed trail. If every two vertices in graph *G*, are the endpoints of a walk, then we say that the graph *G* is connected, else the graph is disconnected and formed by two or more separate sub-graphs called components. When every vertex is adjacent to every other vertex, we have a complete graph, denoted by *K*_*n*_, where *n* is the number of vertices.

**Fig. 2:**
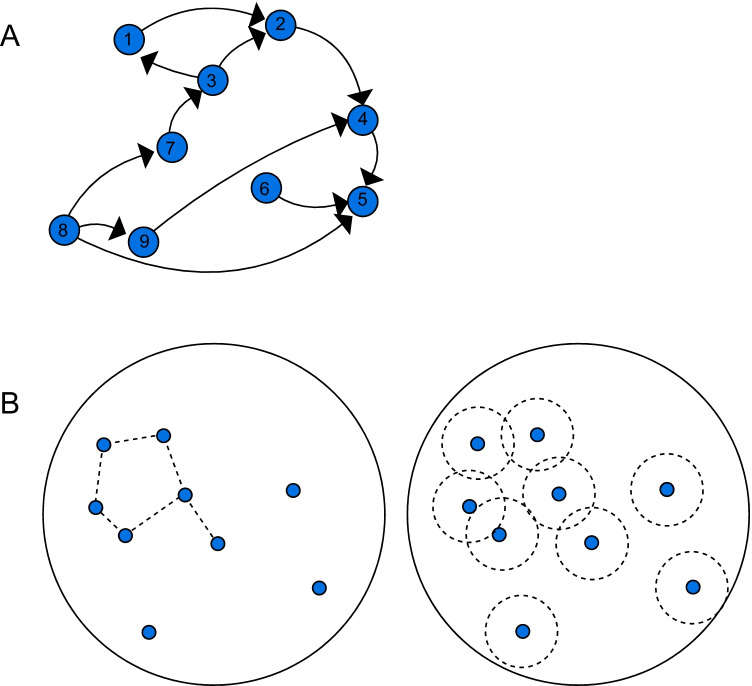
*Top* A small graph on nine vertices connected by a number of directed lines, describing a binary relationship on the set of vertices. Here position in the plane is irrelevant. We will focus on a special case of graphs (random geometric graphs) in which connectivity is related to distance and edges are non-directional. *Bottom* Comparison between the occupancy model and the graph theoretic approach. On the left, the connecting edges are based on a distance threshold, whereas on the right we show overlapping occupancy regions

A simple visual representation of a graph is shown in Fig. 2A. Here edges have a direction. More relevant for us is a type of graph called a random geometric graph. In this case the vertices are placed in a metric space, e.g., the plane, and are connected by an edge if and only if their Euclidian distance is less than a threshold, i.e., a radius *r*. A simple example is shown in Fig. 2B.

## Graph theory and occupancy

Graphs and graph-theoretical measures provide ways to quantify the difference between two configurations. If the visual system is sensitive to these properties, these differences may explain perceived numerosity. A few studies have already employed some of these indices (e.g. Bertamini et al., 2016, 2018; Im et al., 2016), and more recent work has computed average edge length on a nearest-neighbor graph as an alternative to occupancy (Allik & Raidvee, 2021). Unlike other measures such as size of the physical elements, measures based on graph properties are not based on arbitrary units (e.g., pixels, centimeters, inches).

For any 2D configuration, two vertices can be considered connected if their Euclidean distance is less than a given distance *d*, thus defining a random geometric graph *G*_*d*_. Note that this distance is closely related to the region of influence hypothesized in the context of the occupancy model. The overlap between two neighboring regions of influence occurs when the distance between the vertices is no larger than 2*r*, where *r* is the occupancy radius; if we set *d* = 2*r* then the edges of *G*_*d*_ describe exactly those elements whose regions of influence intersect. Figure 2B compares the two approaches for an example with only nine vertices.

**Fig. 3:**
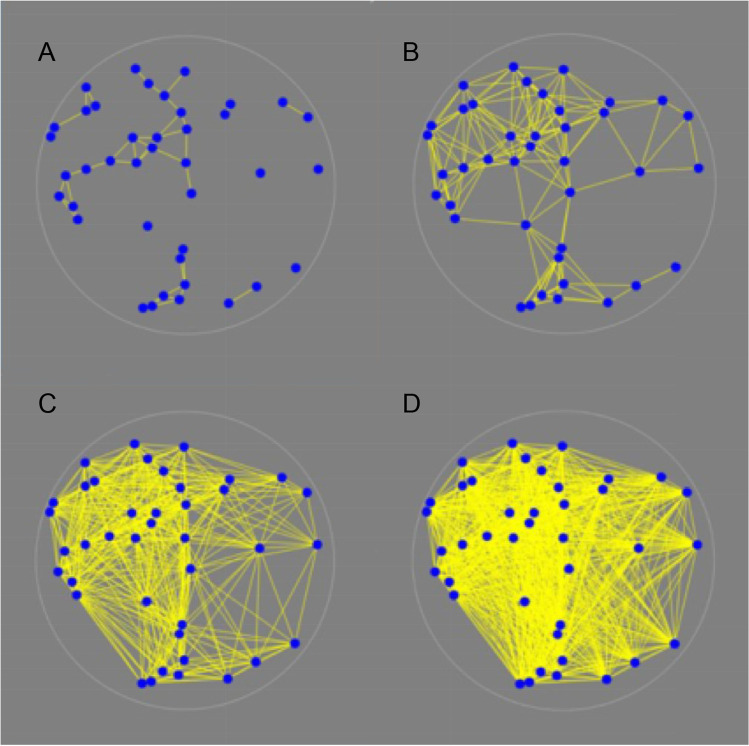
**A** connectivity distance = *R*/4, number of edges 43, the graph is disconnected, **B** connectivity distance = *R*/2, number of edges 171, the graph is now connected. **C** connectivity distance *R*, number of edges 468, the graph is becoming denser. Finally, **D**, connectivity distance 2*R*, number of edges 780, this is the complete graph K_40_

As in the case of the occupancy model, the graph structure varies with the distance parameter, the larger the value of *d* the more edges there will be in *G*_*d*_. Figure 3 shows a random 40-vertex configuration inside a circle *C*_*R*_ of radius *R*. In panel A, at a small connectivity distance of *d* = *R*/4, the graph is disconnected into ten components, the largest of which has 18 vertices. As the connectivity distance increases, eventually the graph connects (panel B). If the connectivity distance increases, more vertices will be connected, and the graph becomes denser. Finally, at *d* = 2*R* all vertices are connected, and the last panel (D) describes the complete graph *K*_*40*_.

The difference in graph structures between panels suggests that many properties of random geometric graphs change with connectivity distance. It is clear from the top left panel in Fig. 3 that a small value of *d*, leads to *G*_*d*_ being split into two or more components. The other three panels represent a connected graph.

There is an important difference between the occupancy model and any index defined on graphs. Graphs are abstract entities defined on vertex configurations on the basis of adjacencies. Even when edges describe geometrical proximity between elements, the existence of the edges of *G*_*d*_ depends on the proximity of the vertices with a strict “all-or-none” criterion.

It is also important to say what aspects we did not consider in this study. We focus on the properties of the configuration, defined by the relationship between locations. The nature of the elements themselves is not relevant. We do acknowledge that numerosity perception is affected by properties of the elements. For example, 20 dots may have twice the surface area of ten dots. The correlation of these continuous measures with numerosity is a problem, recently discussed by Salti et al. (2017). However, these physical properties of the elements are outside the scope of our paper.

Bertamini et al. (2016) used some graph indices in their study of numerosity. They considered three numerical measures of the configurations: the area of the convex hull, total degree, and (average) local clustering. They then compared these measures to predictions made by the occupancy model. The convex hull is a useful measure of overall dispersion. In 2D it can be defined as the closed curve with minimum perimeter containing all elements. Unlike the other indices discussed, it does not depend on the connectivity parameter *d*. The degree of a vertex was defined earlier as the number of its edges, to get the total degree we sum this for all vertices. Because an edge always connects two vertices, total degree is twice the number of edges. Finally, the local clustering index will be defined in the next section. Bertamini et al. (2016) generated datasets for a limited range of connectivity distances *d* (approximately between *d* = *R*/8 and *d* = 3*R*/4, in steps of *R*/16), and then used correlational analysis to compare indices. However, the values of *d* did not cover the full range of possibilities (as shown in Fig. 3, *d* can take on any positive value up to 2*R*).

In this paper, we consider ten graph indices along with the occupancy model and study their correlations for randomly generated vertex configurations. This list is not exhaustive, but it includes well-known indices developed to capture "clustering". As argued in the Introduction, there is evidence pointing to the importance of grouping and clustering (e.g., the Solitaire illusion). Moreover, for comparison we included the occupancy model.

We first investigate correlations between the indices for fixed values of the connectivity distance, across its full range. Certain correlations will depend on the connectivity distance, and in addition, some of the indices will only be valid for a limited range of d. We then describe a strategy that captures the key properties of the correlations between indices. We selected a specific connectivity distance for each index and then correlated the index values obtained at this distance. The correlation results obtained in this way capture the main correlations observed at fixed connectivity distances. Finally, a principal component analysis was performed in an attempt to isolate meaningful clusters of similar indices.

Correlation studies on graph indices have been attempted before on a smaller scale. For instance, Guzman et al. (2014) looked at different types of centrality and clustering measures, for a collection of 320 different graphs (or networks as these were called). In another study, Meghanathan (2016) concentrated on centrality measures and their correlation with maximal clique, with the aim of using correlational analysis to find computationally “light weight” alternatives to maximal clique. We will discuss the definition of clique in the next section.

A correlational analysis enables us to study certain (difficult) graph indices by working with related (simpler) ones. This has been done before, analytically, in several studies of random graph properties. The degree of the vertices in a graph *G* is related to its chromatic number, the minimum positive integer *k* such that the vertices of *G* can be coloured with *k* colors and no pair of vertices connected by an edge receives the same color (Shi & Wormald, 2007). The number of edges affects the presence of global structures, for instance a Hamiltonian cycle, which is a long cycle that visits every vertex of *G* exactly once (Korshunov, 1976). However, this is difficult for the general case. Apart from results in a few classical models of random graphs, only empirical analyses exist on more realistic distributions (Guzman et al., 2014; Meghanathan, 2016).

The remainder of the paper is organized as follows. In the next section, the selected indices are described. We then provide all the details of our tests. The last part of the paper is devoted to an analysis of the results.

## Description of the indices

Hundreds of different graph indices exist. In choosing the list of indices, we picked measures that are sensitive to connectivity distance, and indices that have been studied in other disciplines. We wanted to take advantage of the range of features that different graph indices can compute. For instance, the information spread on graphs can be affected by only a small number of vertices (Karunakaran et al., 2017), and there are many centrality measures that are known to extract information about such vertices. We will work with Eigenvector Centrality as an example of such centrality measures. Eigenvector Centrality is used in social network analysis, as well as clique finding and clustering (Wasserman & Faust, 1994). Both independent and dominating sets have been used in wireless sensor networks (Basagni, 2001; Fu et al., 2015). Lastly, we included connected components and total degree as these are particularly sensitive to groupings and density, which are known to be of importance in studying numerosity (Anobile et al., 2015; Frith & Frith, 1972).

Definitions in each case (except for occupancy) are given in Table 1. The reader is referred to standard textbooks like Harary (1969) for basic graph theoretic definitions. To avoid dealing with range heterogeneity, all indices were normalized by the maximum value for a given fixed number of vertices *n*. However occasionally the normalization factor depends on *d*.

**Table 1:** List of indices, including their acronyms, any normalization requirements and descriptions

Index	Description
Total Degree (TD)	The Total Degree in a graph is the sum of the degrees of its vertices. Each edge is adjacent to two vertices, and the sum of the degree is therefore also twice the number of edges. $\sum\limits_{v\in V}\deg v=2\mid E\mid$ We normalize this value by dividing it by the maximum number of edges possible, *n*(*n* − 1)/2, where *n* denotes numerosity
Total Edge Length (TL)	The Total Edge Length (TL) of a graph is the sum of the Euclidean distance between all pairs of vertices at a distance of at most *d*. $TL=\sum\limits_{\left\{u,v\right\}\in E(G)} dist\left(u,v\right),$ The quantity is normalized by dividing it by 2*d* ∣ *E*(*G*)∣
Random Walk Cover Time (RW)	A random walk on a graph *G* is a process that, starting at an arbitrary vertex, hops around in discrete times steps following random edges. We are interested in the time needed for the walk to visit (at least once) all vertices of *G*. We only consider random walks for graphs that are connected. For each *G*_*d*_ we did 1000 random walks and computed the mean of the number of steps taken. The measure RW is then obtained as the inverse of this number multiplied by its theoretical minimum, which is slightly less than *n* log *n*, where *n* is the numbers of vertices, as proved by Kahn et al. (1989)
Eigenvector Centrality (EG)	Eigenvector centrality gives a value to each vertex, proportional to the sum of the values of its neighbors (Bonacich, 1987). Formally such vector can be obtained as solution of the equation ***Ax*** = *λ****x*** where ***A*** is the adjacency matrix of the given graph, and *λ* its eigenvalue. The vector ***x*** can be computed iteratively, starting by setting ***x*** to be the degree sequence of the given graph. The multiplication ***Ax*** generates a new vector. Matrix ***A*** can be multiplied by such vector and the whole process repeated until the product ***Ax*** stabilizes to satisfy the equation above. If a convergence is achieved after n iterations, then real value *λ* is an eigenvalue of ***A***, and the average value of the components of the resulting ***x*** defines the eigenvector centrality of the given graph. If there is no convergence, then its value is zero. This value is normalized by using the square root of the sum of each eigenvalue, on each iteration
Connected Components (CC)	At low connectivity distance, graphs are typically not connected. They can be described as formed by a number of connected components, each containing at least one vertex. Single vertices are treated as one component, the maximum number of components that a graph can have is the cardinality of the set *V*. As the connectivity distance grows the number of components reduces and eventually *d* is so large that the graph becomes connected. CC is the number of connected components, divided by the number of vertices
Clique Number (CL)	A clique, in a graph *G*, is a subgraph that is itself a complete graph. The size of the largest clique in a graph *G* is called the clique number of *G*. For normalization we divide this index by the number of vertices *n* = ∣ *V*∣
Domination Number (DN)	In a graph *G*, a dominating set *D* is a subset of the vertex set *V*(*G*), such that every vertex in *V*(*G*) is either a member of *D*, or is adjacent to a vertex in *D*. The set *V*(*G*) is trivially a dominating set. The cardinality of the smallest dominating set for *G*, is called the domination number (DN) of graph *G*. The domination number of *G* is then divided by the number of vertices *n* = ∣ *V*∣
Independence Number (IN)	Two vertices in a graph are pairwise independent if they are not connected by an edge. This concept of pairwise independence enables us to study non-trivial, large dominating sets. An independent set in a graph *G* is a set of pairwise independent vertices. Trivially, a set with one vertex is an independent set, and, in general, maximal independent sets are also dominating sets. A maximum independent set (IN) in *G*, is an independent set in *G* of largest cardinality, and its cardinality is the independence number of *G*. The independence number of *G* is then divided by the number of vertices *n* = ∣ *V*∣
Local Clustering Coefficient (LC)	The local clustering coefficient *lc*_*v*_ of a vertex in a graph *G*, calculates how close its neighbours are to form a complete graph (Watts & Strogatz, 1998). If we let number of edges induced by connectivity distance *d*, be *e*(*V*) and the set of vertices connected to a *v* be *N*(*v*), then the local clustering coefficient *lc*_*v*_ can be defined as: ${lc}_v=\frac{2e\left(N(v)\right)}{\deg v\left(\deg v-1\right)}$ The average value is found by summing the coefficient of each vertex, then dividing by *n* = ∣ *V*∣
Global Clustering Coefficient (GC)	The global clustering coefficient (GC) differs from the local version above, in that it attempts to capture the clustering in a graph as a whole, not just a local\neighborhood level. The first attempt to formalize such notion dates back to Luce & Perry (1949). The concept has had a revival of interest at the turn of the century (Wasserman & Faust, 1994) in the context of social network analysis. Its computation involves finding the ratio of closed triplets (3 vertices forming a triangle) and open triplets (two out of three vertices are connected) in *G*. $gc=\frac{3\times Number\ of\ triangles}{Number\ of\ connected\ triplets}$

## Methods

In this paper, we study configurations of elements with four different numerosities {22, 28, 34, 40}, confined to a circular area of radius *R*. These numerosities are above the subitizing range, and unlikely to form a texture. To estimate the density for an observer, we must assume a viewing distance. On a typical computer display at 57 cm, *R* equals 160 pixels if we assume there are 32 pixel/cm. Therefore, densities would be: 0.28, 0.36, 0.43, 0.51 elements/deg^2^, respectively.

One thousand random patterns in the 2D plane were produced for each value of *n*. There are excellent tools for generating patterns (De Marco & Cutini, 2020; Gebuis & Reynvoet, 2011). We opted for a simple rejection sampling technique: select a random location in a rectangle and keep it if it is inside the circle *C*_*R*_ and no other element is within distance *δ* from it. The first constraint limits the vertex spread to a finite circular region. This is useful because in the context of human vision a circular region has a specific level of eccentricity, i.e., distance from fixation. With respect to the second constraint, the parameter *δ* avoids overlap. We used *δ* = *R*/16. Under these conditions the event that two vertices are within a distance of *δ* has a probability of approximately 0.0038. Hence, depending on *n*, few overlaps occur, which in turn motivates the adopted rejection sampling strategy.

## Implementation

An interesting feature of the indices considered in this study is their computational complexity. Some of them (e.g., local or global clustering, connected components, total degrees and total edge length) can be computed in a time that is proportional to the size of the input graph. However, some pose computational problems (eigenvalue centrality and occupancy), and others are slow to compute (clique, and dominating sets). We used Python 3.6 combined with libraries *Networkx v2.5* (Hagberg et al., 2008) and an extension to Networkx; *GrinPy* v19.5a0 Amos, Davila, 2019) for most of the computations (including max cliques, dominating and independent sets). However, we resorted to a more ad hoc method in the case of occupancy, which will be explained next.

To compute occupancy, one needs to find (the proportion of) the total area of interest that falls within at least one of the influence areas. This is an interesting computational problem which has been studied in the past (Edelsbrunner, 1993). Methods exist that compute the occupancy of a set of circles in a time proportional to the number of circles (Aurenhammer, 1988). Such methods are not always useful in practice. Bertamini et al. (2016) used an exhaustive process that calculated the occupancy by computing all possible circle intersections. The properties of those configurations meant that the process could be completed in a reasonable amount of time. In the current study, we compute occupancy values for larger influence radii. When the influence radius becomes large most circles intersect, and the exhaustive process becomes slow. We therefore resorted to an alternative approach. To calculate occupancy, the Python library *PsychoPy* (Peirce, 2009), was used.

**Fig. 4:**
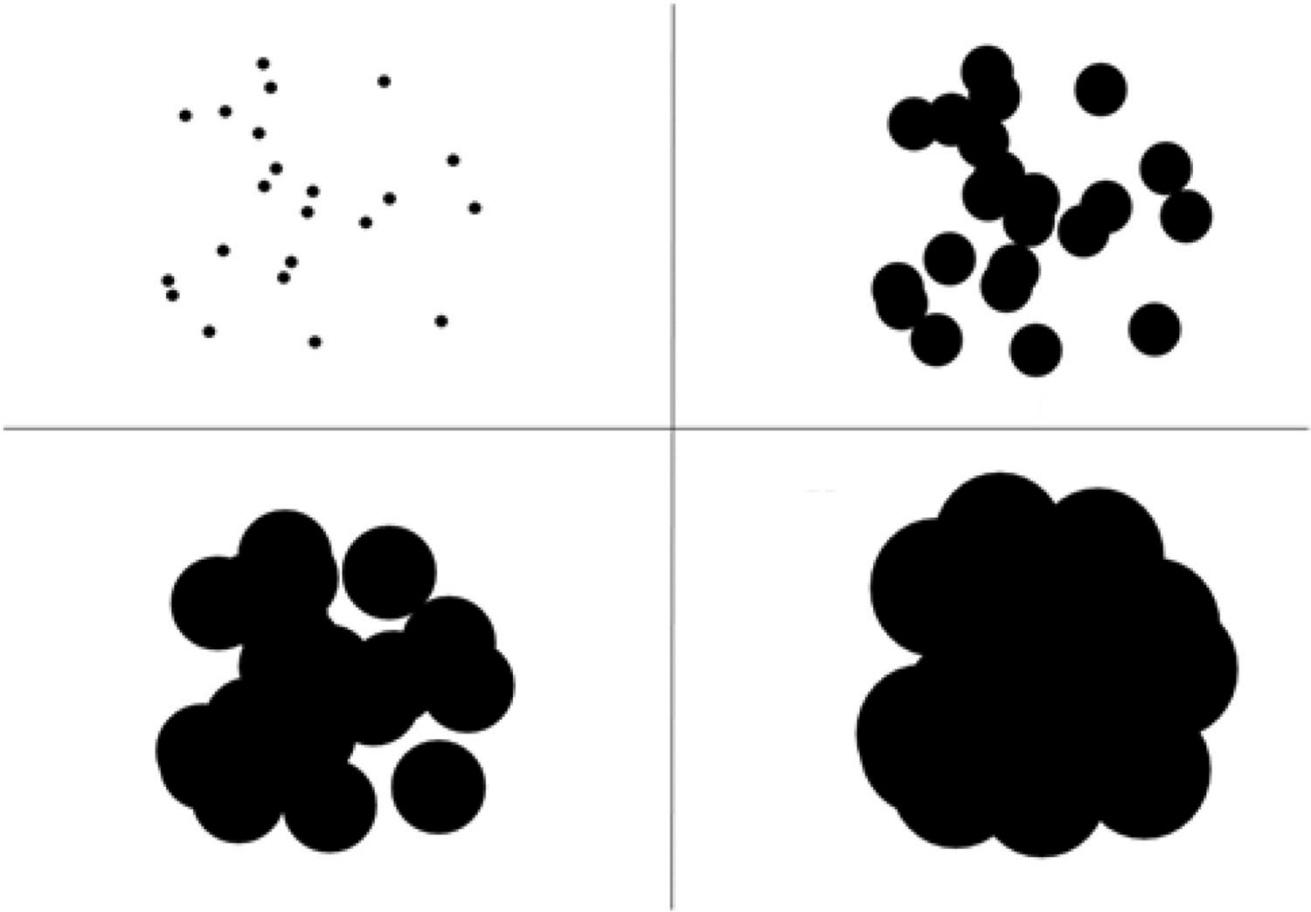
How occupancy value (OC) changes with an increase in the center-to-center distance d. Top left *d* = *R*/16, top right *d* = *R*/3, *bottom left d* = *R*/2 and *bottom right d* = *R*. For all patterns numerosity is 22

For each point, a black circle of radius *r* was drawn on a white background, see Fig. 4 for example of a configuration for *n* = 22, for various occupancy radii. The PsychoPy function *getMovieFrame()* then captured the image of the screen, after which the number of black pixels counted. Hence the algorithm returns the total number of black pixels, normalized to the maximum possible value that the occupancy can take, which is nπ(*d*/2)^2^, the area of n disjoint circles of radius *d*/2. The method was tested against the exact algorithm used by (Bertamini et al., 2016) and found to have correlations larger than 0.998 across *d* = *δ* to 12*δ* pixels, in steps of *δ*. Hence, although this algorithm is an approximation, it still produces a valid measure of occupancy. We used the library *Networkx* for finding the average eigenvector centrality (cf. Guzman et al., 2014). To ensure convergence, we used 1000 iterations for each *G*_*d*_.

Values for all indices are made available on Open Science Framework: https://osf.io/yxdvm/. In addition, we made available a full set of Python functions that can analyze any set of coordinates; these functions are provided with documentation and examples.

Researchers can specify which index to compute and over what connectivity distance range. See the Python script “*run_me.py*”, on how to input any number of dot patterns, and subsequently compute either occupancy values, or one of the graph indices described in this work. When executed, the script returns an excel dataset consisting of the chosen index, computed over each value of *d* and for each dot pattern. This script also prints out the value of *d* that gives the maximum standard deviation.

It is possible to input just one pattern and use that to compute different indices over different values of *d*. See the script “*run_me_toy_example.py”*, for ways to draw graphs, and visualize indices such as independence number, and local clustering coefficient, using the drawing package provided by the library *Networkx*. Using these scripts, researchers can input one dot pattern at a time and “play” with a graph index over any chosen range of *d*. There is an accompanying document, called “InstructionManual.pdf” that describes each of the steps taken in “*run_me.py*”, and “*run_me_toy_example.py”*. The document also demonstrates how to create visual illustrations and heatmaps similar to those used in this paper.

## Correlational analysis results

**Fig. 5:**
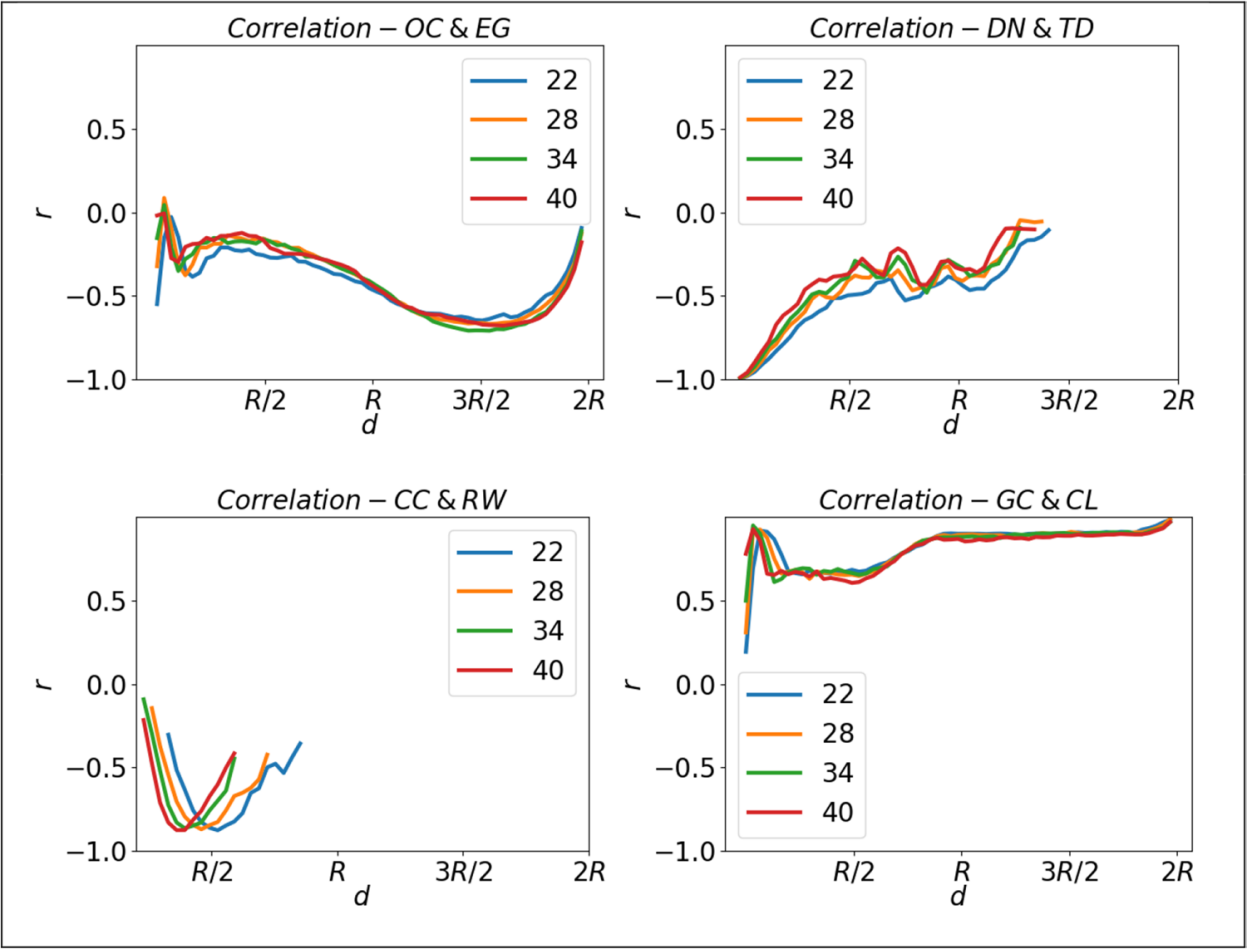
All correlations computed using the Pearson coefficient, any with *p* > 0.05 were ignored. *Top* and *bottom left* show examples of transient correlations. *Top left* EG and OC are strongly correlated *r*(998) >  ∣ 0.6∣, at 3*R*/2, but much lower for other *d* values. Also, CC and RW are strongly negatively correlated for *d* = *R*/2, but the correlation decreases rapidly elsewhere. *Bottom right* shows both CL and GC are strongly positively correlated, throughout the full range of *d*. *Top right* shows how some indices will have a threshold value, DN hits it minimum value across all *n* when *d* = 3*R*/2

We computed the correlation matrices between indices at each *d* ∈ {δ, δ + 5, δ + 10, δ + 15, …, 2*R*}. This in turn was repeated for each value of *n*. The resulting correlational matrices revealed the presence of transient correlations (i.e., correlations between pairs of indices that only existed at a particular connectivity distance, see Fig. 5) and also pathological distributional patterns in the underlying data.

The top and bottom left panels of Fig. 5 show how, over a small range of *d*, indices can be correlated, for instance CC and RW are strongly negatively correlated only in a range around *d* = *R*/2. In the bottom right panel, CL and GC formed strongly positive correlations across most of the range of *d*. It is also clear from the top right and bottom left panels of Fig. 5, that indices may reach their respective limiting values at different *d*. As an example, the threshold values for CC (bottom left panel of Fig. 5) are 22: 0.75R, 28: 0.625R, 34: 0.625R and 40: 0.56R. Values greater than this for *d* will guarantee all graphs are connected, and hence the variance in CC will be zero. Likewise, DN reached its threshold value for all *n* by *d* = 3*R*/2.

**Fig. 6:**
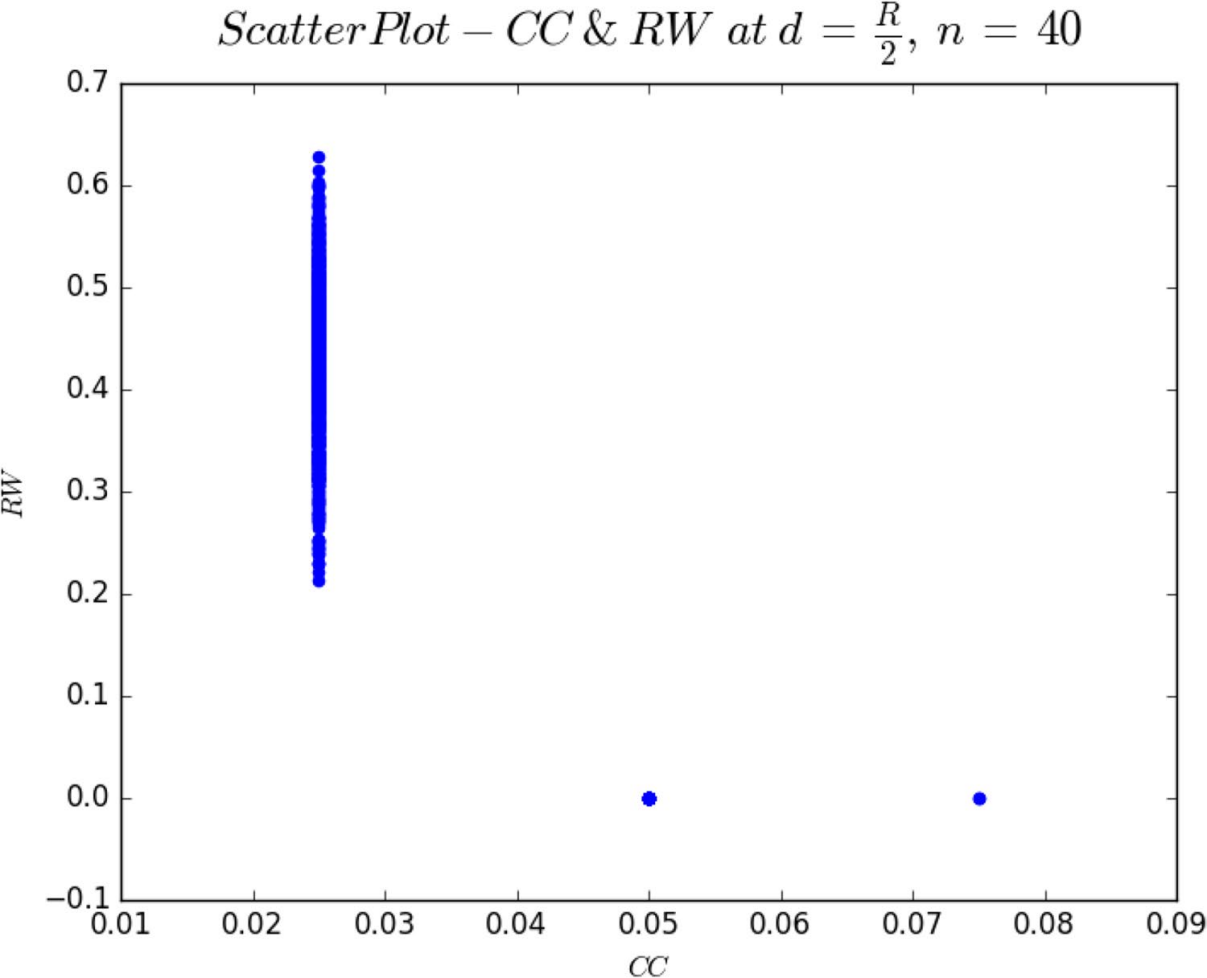
Scatter plot of connected components against random walk at connectivity distance *R*/2. This has a correlation of *r*(998) =  − 0.849, *p* = 0.000. The strength of the correlation is due to CC approaching its threshold value, and random walk becoming a computable index

Indices approaching threshold values raise problems with computing correlations. For instance, when we investigated the maximum correlation for RW and CC, Fig. 5 bottom left panel, the correlation between CC and the RW at *n* = 40 and *d* = *R*/2 was *r*(998) =  − 0.816, *p* = 0.000. Figure 6 shows a scatter plot of RW and CC at *n* = 40, and *d* = *R*/2. It is clear that the relationship between CC & RW is not linear.

The root of the problem is that CC is getting close to its threshold value as most graphs start to connect, while RW is only just becoming a computable index for the same reason. When plotted, CC is oscillating between to two values, and RW has values that are zero and thus acting as outliers. Each correlation matrix we wish to compute requires 45 separate correlation computations, at each data point in *d*. Hence inspecting each correlation visually would not be feasible. The problem is compounded by a sample size of *N* = 1000. Tests for normality, for instance Kolmogorov–Smirnov and Shapiro–Wilk, are known not to be accurate for *N* > 300 (see Field, 2009, for discussion). Nevertheless, Fig. 6 does suggest that these cases do not contain enough variability at a specific value of *d*. The ability to filter out unreliable correlations will be the topic of the next section.

When we iterated through *d* we found two groups of strongly correlated indices forming across all *n*. From now on we refer to indices OC, DN, IN, and CC as belonging to the clustering group, and CL, TD, and TL as belonging to the spread group. We found other correlations, however these were either expected, such LC and GC, or artificial correlations due to indices converging on the same threshold value when the graphs started to become dense.

**Fig. 7:**
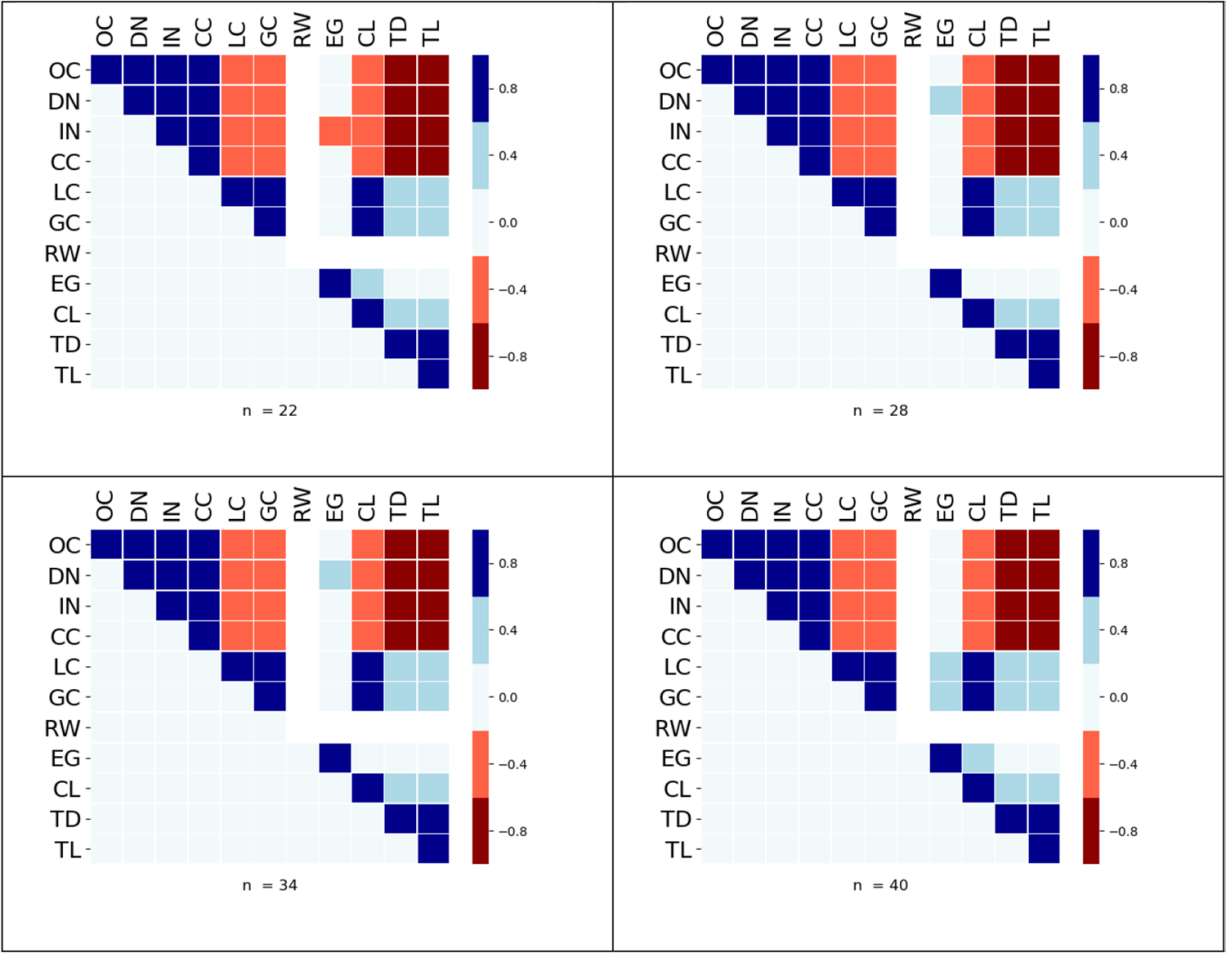
Heat maps for *d* = *R*/8 we see clear patterns emerging that are independent of *n*, for instance the positively correlated cluster of OC, DN, IN & CC

Heat maps for connectivity distance *d* = *R*/8 are shown in Fig. 7, for each *n*. It is clear the clustering group is present, colored blue, top left. We would expect both DN and IN to correlate strongly with each other as both search for a dominating set, in the case of DN it searches for one with a minimum cardinality, whereas IN searches for one with a maximum cardinality. Of more interest is that they also cluster with CC and OC. In addition, TD and TL formed a strong negative correlation with the clustering group. This group does not persist across a wide range of *d*, as CC no longer varies after graphs become connected.

**Fig. 8:**
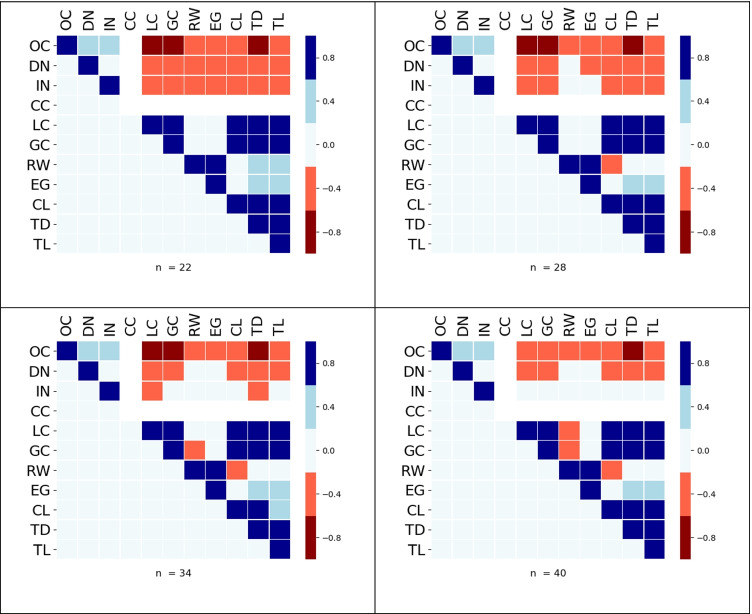
Heat maps for *d* = *R*. Again, clear patterns emerge that are independent of *n*, notably a cluster of highly positive correlations between TL, TD, and CL

Figure 8 shows the heat maps at *d* = *R* for all *n*. As this is the radius of the enclosing circle *R*, all graphs will be connected with a high probability, consequently the index CC will have reached its threshold value, and its variance will be zero. Therefore, the clustering group has disappeared, but the spread group has formed for all *n*. Also, LC and GC have now strong correlations with the spread group.

## Comparisons based on maximum standard deviation

The analyses presented for each *n*, enable us to obtain correlation matrices at each *d* ∈ {δ, δ + 5, δ + 10, δ + 15, …, 2*R*}. We reasoned that it would be useful to find where indices are most informative, select only one value of *d* per index, and then show how indices relate to each other when computed on graphs obtained by using those particular values of *d*.

In an attempt to capture the most significant correlations, we computed, for each index *m*, the value *d*_*m*_(*n*) of the connectivity distance that maximizes the standard deviation *σ*_*m*_(*n*, *d*), and then studied the correlations between the values of the 11 indices computed in the graphs ${G}_{d_m(n)}$. We call these the maximum standard deviation correlations. Table 2 shows the values of *d*_*m*_(*n*) for all indices.

**Table 2:** Each cell displays the numerical value computed for the maximum standard deviation for each index, and the same value in terms of *R, column N is numerosity*. The value of 160 for *R* was chosen because this would correspond to 5^°^of visual angle on a screen at distance 57 cm (assuming 32 pixels/degree)

N	OC	DN	IN	CC	LC	GC	RW	EG	CL	TD	TL
**22**	60 0.38R	30 0.19R	35 0.22R	35 0.22R	55 0.34R	55 0.34R	125 0.78R	70 0.43R	220 1.38R	180 1.13R	155 0.97R
**28**	55 0.34R	30 0.19R	30 0.19R	35 0.22R	50 0.31R	45 0.28R	115 0.72R	65 0.41R	230 1.44R	170 1.06R	155 0.97R
**34**	50 0.31R	30 0.19R	25 0.16R	30 0.19R	45 0.28R	40 0.25R	105 0.66R	65 0.41R	235 1.47R	170 1.06R	160 R
**40**	45 0.28R	25 0.12R	25 0.16R	25 0.16R	40 0.25R	35 0.22R	95 0.59R	60 0.38R	240 1.5R	175 1.09R	160 R

**Fig. 9:**
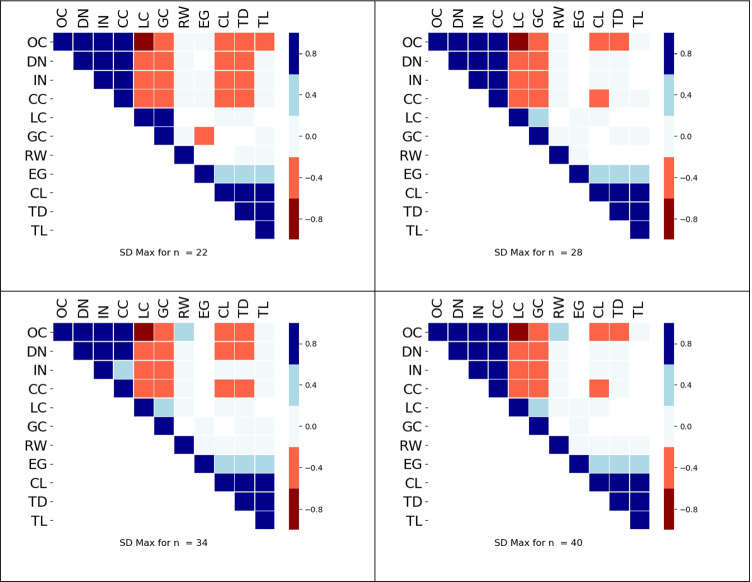
Heat maps with correlations between indices selected based on standard deviations. We see that the use of SD Max has captured the clustering and spread groups

This analysis for the maximum standard deviations was repeated for each index and the heat maps are shown in Fig. 9. Notice that the two most interesting components seen in the correlational analysis between measures at fixed values of *d* are still present. We see a strong negative correlation between LC and occupancy. Also, EG is strongly correlated with the spread group, even though the computed value of *d*_*EG*_(*n*) is closer to the average value of *d* for the clustering group.

It might be argued that correlating the 11 indices using the values obtained in ${G}_{d_m(n)}$ in each case is artificial, as we are comparing measures obtained from different graphs, due to the different connectivity distances. However, the graphs we use are not totally unrelated. As we pointed out in the Introduction, the geometric graphs we are working with are such that if *d*_1_ ≤ *d*_2_ then ${G}_{d_1}$ is a subgraph of ${G}_{d_2}$. If ${d}_{m_1}(n)<{d}_{m_2}(n)$. comparing *m*_1_ in ${G}_{d_{m_1}(n)}$ with index *m*_2_ in (the denser) ${G}_{d_{m_2}(n)}$ may ignore irrelevant information about ${G}_{d_{m_2}(n)}$ when dealing with *m*_1_. The goal is to find graph-theoretic indices that predict human numerosity judgements. These indices depend on the set of vertices but also on the value of *d*. Therefore, comparing indices using the same value of *d* might not be ideal.

## Principal component analysis

We investigated the maximum standard deviation correlation matrices further, by performing a principal component analysis (PCA), with the purpose of extracting any uncorrelated\orthogonal features that are present in the data. We omitted the index RW as it is clear from the previous section that it did not correlate with any other index. Please note that the term “component” as used in PCA is not the same as a “component” in graph theory. SPSS confirmed two strong eigenvalues, and a third just below 1, from the covariance matrix of *σ*_*m*_(*n*, *d*). We summarize the results in Table 3.

**Table 3:** Summary of the first two eigenvalues found from the PCA analysis and cumulative variance explained from their components for each *n*. We see most of the variance is explained by these first two components

	*n* = 22		*n* = 28		*n* = 34		*n* = 40	
	Eigenvalue	Cumulative variance (%)	Eigenvalue	Cumulative variance (%)	Eigenvalue	Cumulative variance (%)	Eigenvalue	Cumulative variance (%)
Component 1	4.58	45.75	4.01	40.54	4.22	42.18	4.26	42.60
Component 2	2.8	73.44	2.9	69.53	2.72	69.34	2.90	71.56

The varimax matrix extracted two components, with the following members: component 1: OC, DN, IN, CC, LC, and GC, and component 2 containing EG, CL, TD, and TL. Largely confirming the two groups (clustering, spread) seen in the informal correlational analysis in the previous section. Also, the PCA placed LC inside PCA component 1 with a load factor ranging between – 0.76 and – 0.70 dependent on *n*. This was reflected in the correlational analysis at fixed distances, since it found negative correlation values < − 0.3 between LC and OC until LC started reaching its threshold value as the graphs became dense. Using the same argument, GC was consistently moderately negatively correlated > − 0.6 and < − 0.2 with OC, which is reflected in its load factor between – 0.68 and – 0.48 (see Table 4).

**Table 4:** Rotated matrices produced by a PCA. Two components are clearly seen across all densities. Note that loadings below, values of .3 or less have been suppressed

*n* = 22	Component		*n* = 28	Component	
	1	2		1	2
OC	*0.90*		OC	*0.92*	
DN	*0.85*		DN	*0.90*	
IN	*0.83*		IN	*0.83*	
CC	*0.90*		CC	*0.83*	
LC	*– 0.76*		LC	*– 0.72*	
GC	*– 0.68*		GC	*– 0.53*	
EG		*0.75*	EG		*0.63*
CL		*0.86*	CL		*0.88*
TD		*0.94*	TD		*0.96*
TL		*0.91*	TL		*0.92*
*n* = 34	Component		*n* = 40	Component	
	1	2		1	2
OC	*0.90*		OC	*0.90*	
DN	*0.90*		DN	*0.93*	
IN	*0.75*		IN	*0.86*	
CC	*0.90*		CC	*0.95*	
LC	*– 0.73*		LC	*– 0.70*	
GC	*– 0.48*		GC	*– 0.51*	
EG		*0.67*	EG		*0.67*
CL		*0.86*	CL		*0.87*
TD		*0.96*	TD		*0.96*
TL		*0.93*	TL		*0.94*

For completeness, it should be noted that there are other pairwise features between indices that are not present in either correlational or principal component analysis. For instance, GC and CL were strongly correlated through a wide range of values for *d*, as were OC and TD. This is not seen in either the correlational analysis or PCA of the maximum standard deviations (this could be due to their respective *σ*_*max*_ being far apart in terms of *d*, as seen in Table 2). In Fig. 7, we also notice a strong correlation between the spread group and indices LC and GC. This also has not been extracted. However, correlations between the spread group and indices {LC, GC} were more transient and relied more heavily on connectivity distance *d*.

## Discussion

This study had three goals. The first was finding useful graph indices for the study of numerosity. We wanted to expand on the approach taken by some researchers in the numerosity literature who used graph indices to predict human and animal numerosity perception (Bertamini et al., 2016; Im et al., 2016). For this reason, we selected a list of graph theoretic indices that might be useful in the study of numerosity estimation. Since many numerosity studies involve the use of random configurations of elements as stimuli, we focused on random geometric graphs for our analysis, as they are determined by the geometric distribution of a configuration, combined with a connectivity distance parameter. This approach enabled the more formal study of the combinatorial/geometric properties of this type of stimuli. It was important, in choosing indices, that the graph properties they measured were wide-ranging, in the sense that we would have indices sensitive to groupings/clustering, density, and other graph properties such as centrality and the cardinality of the independent/dominating set.

We analyzed ten indices: number of connected components CC, domination number DN, independence number IN, average local clustering coefficient LC, global clustering coefficient GC, average eigenvector centrality EG, random walk RW, maximum clique size CL total edge length TL and total degree TD. Together these indices represent a broad range of properties found on graphs. The correlational analysis also enabled us to study certain (computationally intractable) graph indices by working with related (simpler) ones.

The second aim followed directly from the first one. We wanted to compare the graph theoretic approach with the occupancy model (Allïk & Tuulmets, 1991). We therefore added this measure (denoted by the abbreviation OC) to the ten mentioned above, thus providing a total of eleven indices. Although there is a relationship between connectivity distance of random graphs and the occupancy radius, OC represents a fundamentally different way to capture clustering and grouping properties. Edges on graphs represent relationships between elements (an all-or-none relationship), whereas occupancy is based on the idea that each element has a region of influence, estimated by a circular area. The total area of influence is then taken as the predictor of the overall perceived numerosity. There is a parallel between the connectivity distance used to construct a graph, and the size of the region of influence, and therefore we manipulated this factor in a similar way for all measures (that is, distance affects both edge creation between two vertices on a graph, and the overlap of each elements region of influence). The results indicate a strong correlation between occupancy and some other measures, highlighting a couple of important geometric features that affect the perception of numerosity.

Our third aim was to study the correlations between indices, and across a range of connectivity distances. In previous studies, only a subset of values was used (Bertamini et al., 2016). We wanted to see which indices grouped together, and whether these groups would persist across connectivity distances. Finally, we aimed to summarize these results, and draw conclusions about their implications for research in numerosity perception.

The correlational analysis identified many pairwise relationships between individual indices that were transient in nature. Nonetheless, some structures of the pairwise correlational patterns persisted across numerosity, and hence our attention turned towards the formation of persistent groups of indices (three or more highly correlated indices) over specific ranges of the connectivity parameter *d*. We identified two such groups. The first of these (referred to as the clustering group) included OC; IN; DN; and CC. This group had connectivity values around *d* = *R*/8 for all *n*, as shown in Fig. 7. As its connectivity distance was small, it is sensitive to how groups of elements cluster together. The second group (referred to as the spread group) included CL, TL, and TD. This group formed at a larger connectivity distance *d* ≥ *R*, again this was independent of *n*. We felt that at larger connectivity distances this group was picking up on the more global properties of the patterns, like areas of highest density. Of the two groups, the spread group persisted over a larger range of values of *d*. This was because the clustering group contained both CC and DN, two indices whose extreme values are reached well before the other indices.

In an attempt to filter out pathological and transient correlations, and summarize the main results found in the correlational data, four additional datasets were created, one for each value *n*. Each dataset consisted of the value of *d* that generated the maximum standard deviation for each index^1^. When the correlational analysis was repeated on the resulting four datasets, it had the advantage of ignoring trivial and transient correlations. Importantly, it found both groups of indices described in the previous paragraph (Fig. 9).

The maximum standard deviation datasets were investigated further with a principal component analysis, which confirmed that the two groups were orthogonal. Within the first PCA component indices LC and GC joined the group with members OC, DN, IN, and CC. This confirmed our hypothesis that this group of indices is more sensitive to how groups of elements cluster together. The second PCA component included CL, TD, TL, and added EG. Again, the addition of EG makes sense as this index extracts the most influential vertices in graphs. RW was not a member of either components, which reflects the fact that it rarely formed any significant pairwise correlation with another index, other than EG and CL, across any value of *d* or *n*.

The connectivity distance for the clustering group had an average value of *d* ≈ *R*/4, and we know from the correlational analysis that this is below the threshold value for CC. Therefore, the input graphs to the clustering group will be disconnected into discrete units. OC was also a member of this group, and this is consistent with finding that the region of influence operates over a small distance (Allïk & Tuulmets, 1991; Bertamini et al., 2016). Also, Allik and Tuulmet suggested that the optimum value for the occupancy radius may be a property of the type of stimulus. The value that gives the maximum variance could be that property. As an index OC is sensitive to patterns that manipulate the spacing between elements. However, when used on purely random patterns, with no manipulation on spacing, it is also sensitive to groupings. This is confirmed by its strong correlation with CC, something that also became apparent from the PCA. Hence, we suggest that CC could be a computationally efficient alternative to OC for researchers in numerosity.

The spread group had a much larger connectivity distance that was above the threshold value for CC, thus ensuring that the input graphs are connected into one graph component, and hence this group is more sensitive to global structures such as areas of dense clustering (TD, CL), and influential vertices that are central to information spread - EG. It is known that, in enumeration studies, element saliency is important in predicting initial eye fixations and scanning strategies (Paul et al., 2017). Furthermore, in a recent study it was found that centroid measures were most useful in predicting the position of the first eye fixation (Paul et al., 2020). However, such centroid measures use center of mass calculations that will not necessarily return the coordinates of an element. Centrality measures from graph theory, such as EG, can extract features in patterns such as the most influential or salient elements, and thus may provide a more precise method in predicting initial eye fixations. This has been done before: in eye-tracking studies centrality has been shown to provide an effective method of distinguishing facial scan patterns between autistic and non-autistic children (Guillon et al., 2015; Sadria et al., 2019).

Our findings of two components (clustering, spread), is also related to work by Salti et al. (2017). They describe the existence of two categories of continuous magnitudes (intrinsic and extrinsic). Intrinsic relates to magnitudes that can be computed on individual dots, such as total circumference, total area, and average diameter. Extrinsic magnitudes are concerned with indices that are computed on the array, such as convex hull and density. In our work density sensitive indices such as total degree and maximum clique are members of the spread group, and the occupancy model (related to area of influence) is a member of the clustering group. However, for Salti et al. (2017) the location of the elements is only needed for the convex hull. All other indices strongly correlate with numerosity. Recently, De Marco and Cutini (2020) described a novel way of computing density: the length of the shortest path connecting all elements *n*, divided by *n*-1. They note that this measure is negatively correlated with numerosity.

In contrast to these continuous measures, all indices in our approach operate on a discrete structure and are sensitive to the spatial relationships of the elements. A graph G has a finite number of vertices V and edges E, and this approach may have an advantage when representing the configuration of discrete elements, such as an array of dots. For instance, we used total degree TD as a measure of density, which is computationally efficient because it needs only the count of the edges present in a graph, see Table 1. Unlike average distance between elements, TD is positively correlated with numerosity.

## Conclusion

In this paper we describe random 2D configurations with indices based on graph theory, and compare them with the occupancy model. We found that the indices have different properties and are sensitive to different aspects of clustering. Some may be interchangeable because they are highly correlated, potentially providing efficient alternatives to more computationally intensive methods such as the Occupancy index. The analysis of the pattern of correlations suggests two main groups of measures. The first is sensitive to presence of local clustering of elements, the second seems more sensitive to density and how information spreads in graphs. Empirical work on perception of numerosity may benefit from comparing, or controlling for, these properties.

## Appendix

### Glossary

**Approximate number system (ANS)**: Humans and other animals can estimate the size of a set of elements without relying on counting, language or symbols. The system that supports this estimation process has been called the approximate number system (ANS), and sensitivity to differences in numerosity follows Weber's law. It has also been found that ANS performance correlates with mathematics skill.

**Clique**: A (loop-less, simple) graph *G* is a clique (or a complete graph) if every pair of vertices in *G* forms an edge.

**Convex hull:** In the case of geometric elements in the 2D Euclidean plane, the boundary of the convex hull is the simple closed curve with minimum perimeter containing all elements. Visually, one may imagine stretching a rubber band so that it surrounds all the elements. Often what is computed is the total area of the convex hull.

**Degree**: (of a node *v* in a graph) is the number of edges containing *v*. This is usually denoted as deg_*G*_*v* or simply deg*v* when the graph is clear from the context. The sum of all values of Degree is called Total Degree.

**Edge**: see Graph

**Eigenvector:** In linear algebra, an eigenvector ***x*** of a linear transformation *A* is a non-zero vector (a finite sequence of numbers) that changes at most by a scalar factor *λ* when the given linear transformation is applied to it. In symbols *A* ∙ ***x*** = *λ* ∙ ***x***.

**Global clustering coefficient**: the global clustering coefficient in *G* is the ratio

$$\frac{3\times Number\ of\ triangles}{Number\ of\ connected\ triplets}$$

where a triangle is a collection of three edges of the form: (*u*, *v*), (*v*, *w*), (*u*, *w*), and a connected triple is a triplet of vertices *u*, *v*, *w* for which there exist two edges of the form (*u*, *v*), (*v*, *w*).

**Graph**: In the context of graph theory, a graph is a formal structure defined by a set of points (or nodes, vertices) and a collection of unordered pairs of points called edges (or lines, links). The graph is loop-less if it contains no edge of the form (*v*, *v*). The graph is simple if it contains no repeated edge.

**Graph theory**: the study of graphs, which are mathematical structures used to model pairwise relations between elements. Graph theory is part of discrete mathematics.

**Königsberg bridges problem**: Königsberg (now Kaliningrad, Russia) is set on the Pregel River, and has two large island and seven bridges. The problem is to devise a walk that would cross all seven bridges once and only once. This problem has a place in the history of mathematics. In 1736 Leonhard Euler provided a solution (showing that it is not possible) that laid the foundations of graph theory.

**Local clustering coefficient**: The local clustering coefficient *lc*_*v*_ of a vertex *v* in a loopless, simple graph *G* is the ratio

$${lc}_v=\frac{2e\left(N(v)\right)}{\deg v\left(\deg v-1\right)}$$

where *N*(*V*) and the set of vertices connected to a *v* through an edge, and *e*(*X*) is the number of edges connecting two elements of the vertex set X. The average value of *lc*_*v*_ defines the local clustering coefficient of *G*.

**Loop**: In graph theory, a loop is an edge that connects a vertex to itself, (*v*, *v*).

**Node**: see Graph

**Numerosity**: although in English this noun can also mean the state of being numerous, i.e., numerousness, in the scientific study of numerosity it is used to refer to the quantity itself. Therefore, in this sense it is synonymous with what in mathematics is called the Cardinality of a set: a measure of the number of elements of the set.

**Principal Component Analysis (PCA)**: a multivariate data analysis technique that allows to summarize information by means of a small set of summary indices. Statistically, PCA finds hyper-planes in the n-dimensional space that approximate the data as well as possible in the least squares sense.

**Random Geometric Graph:** a particular type of graph. It is an undirected graph constructed by randomly placing vertices in a metric space, i.e., 2D space. The placement is typically random, but the probability distribution can vary. Vertices are connected by an edge if and only if their Euclidian distance is less than a threshold, i.e., a radius *r*.

**Random walk**: in the mathematical sense, a random walk is a random process that generates a path between elements or locations in some mathematical space. The random walk is a sequence of random steps. We are interested in random walks within graphs.

**Subgraph**: (of a graph *G*) is a graph having all its vertices and edges in *G*.

**Subitization**: a rapid and accurate judgment of numerosity performed for small numbers. The term was coined by Kaufman et al. (1949) from the Latin adjective subitus (meaning "sudden"). The term captures a sense of immediately knowing how many items are present in the visual scene. The subtilization range does not exceed 4 or 5 items.

## Footnotes

1 For two of the indices (RW and EG) the value of d that gave the maximum standard deviation was pathological, with most of the values in the data zero, and the remaining values close or equal to 1. Instead, that value of *d* was used that gave the maximum standard deviation, provided more than 99% of the data was non-zero.
